# Luteolin exerts a marked antitumor effect in cMet-overexpressing patient-derived tumor xenograft models of gastric cancer

**DOI:** 10.1186/s12967-015-0398-z

**Published:** 2015-02-01

**Authors:** Jun Lu, Guangliang Li, Kuifeng He, Weiqin Jiang, Cong Xu, Zhongqi Li, Haohao Wang, Weibin Wang, Haiyong Wang, Xiaodong Teng, Lisong Teng

**Affiliations:** Department of Surgical Oncology, The 1st Affiliated Hospital, School of Medicine, Zhejiang University, NO. 79 Qingchun Road, Hangzhou, 310003 Zhejiang China; Department of Pathology, The 1st Affiliated Hospital, School of Medicine, Zhejiang University, NO. 79 Qingchun Road, Hangzhou, 310003 Zhejiang China

**Keywords:** Luteolin, cMet-overexpressing, Gastric cancer, Patient-derived tumor xenografts

## Abstract

**Background:**

Aberrated activation of cMet in gastric cancer contributes to tumor growth, angiogenesis and metastasis. cMet-overexpressing gastric cancer has a poor prognosis because of high tumor metastasis and limited therapeutic options. Luteolin is a common dietary flavonoid with antitumor properties. However, the antitumor effect of luteolin on cMet-overexpressing gastric cancer remain unclear.

**Methods:**

Two cMet-overexpressing patient-derived human tumor xenograft (PDTX) models of gastric cancer were established, and treated with luteolin or vehicle to evaluate the antitumor effects of luteolin. Tumor specimens were subjected to H&E staining and immunohistochemistry. MKN45 and SGC7901 cells that show high cMet expression were treated with varying concentrations of luteolin and evaluated by western blot, cell viability, apoptosis, migration, and invasion assays.

**Results:**

Luteolin inhibited the tumor growth in cMet-overexpressing PDTX models. Immunohistochemistry demonstrated that expression of cMet, MMP9 and Ki-67 were significantly down-regulated. Luteolin inhibited proliferation, promoted apoptosis and reduced the invasiveness of MKN45 and SGC7901 cells. Western blot revealed that luteolin promoted the activation of apoptosis-related proteins, caspase-3 and PARP-1, and down-regulated the invasion-associated protein, MMP9. Further studies demonstrated that luteolin decreased the expression and phosphorylation of cMet, and downstream phosphorylation of Akt and ERK. In addition, luteolin down-regulated phosphorylated Akt independently of cMet. Blocking Akt and/or ERK with the PI3K inhibitor, LY294002, or the ERK inhibitor, PD98059, induced down-regulation of MMP9 and up-regulation of cleaved caspase-3 and PARP-1, resembling the effects of luteolin.

**Conclusions:**

Our findings ,for the first time, demonstrate that luteolin exerts marked antitumor effects in cMet-overexpressing PDTX models of gastric cancer, through a mechanism likely involving cMet/Akt/ERK signaling. These findings indicate that luteolin may act as a potential therapeutic option for cMet-overexpressing gastric cancer.

**Electronic supplementary material:**

The online version of this article (doi:10.1186/s12967-015-0398-z) contains supplementary material, which is available to authorized users.

## Introduction

Gastric cancer (GC) is the one of the most commonly diagnosed cancers, and the second leading cause of cancer deaths worldwide [1,2]. Despite improvements in surgery and chemotherapy, the prognosis of advanced gastric cancer remains poor. cMet is a member of the receptor tyrosine kinase family, and the major signaling cascades activated by cMet include the phosphoinositide 3-kinase (PI3K)-Akt and Ras-mitogen-activated protein kinase (MAPK) pathways that are associated with tumor survival, growth, angiogenesis and metastasis [3,4]. cMet-overexpressing gastric cancer, which accounts for approximately 40% of all gastric cancer cases, has been shown to correlate with an advanced disease stage and poor prognosis [5-7]. Previous studies of gastric cancer have revealed that co-expression of hepatocyte growth factor (HGF) and c-Met has the potential to promote peritoneal dissemination, and that a high level of c-Met expression is involved in the mechanisms of liver metastasis [3,8]. Moreover, cMet-overexpressing gastric cancer cells can acquire resistance to therapy targeted against the HER family, such as epidermal growth factor receptor-2 (Her2) and the epidermal growth factor receptor (EGFR) [9,10]. cMet-overexpressing gastric cancer possesses a more aggressive cancer phenotype and has a poorer prognosis; therefore, optimizing drugs for the treatment of this type of gastric cancer is crucial.

Luteolin (3′,4′,5,7-tetrahydroxyflavone) is one of the most common flavonoids found in various types of vegetables and fruits, such as celery, green peppers, carrots and olive oil. Luteolin shows strong anti-proliferative activity against a diversity of cancer cells, including breast, prostate and gastric cancers [11-13].

Previous studies have indicated that luteolin exerts its anti-tumor actions by affecting numerous biochemical pathways critical for the regulation of cell survival, apoptosis, angiogenesis and metastasis, including PI3K/Akt, nuclear factor-κB (NF-κB), MAPKs, matrix metalloproteinases (MMPs) and E-cadherin [14-18]. In addition, recent experimental studies have shown that luteolin can suppress HGF-induced c-Met phosphorylation in HepG2 cells, and inhibit the expression of cMet in DU145 prostate cancer cells [8,19]. Although it has been suggested that luteolin possesses strong antitumor characteristics, an effect on cMet-overexpressing gastric cancer cells has yet to be clearly demonstrated.

One of the main obstacles hampering progress in oncological drug research is a lack of appropriate preclinical models. Patient-derived human tumor xenograft (PDTX) models, which closely retain the histopathologic, genetic and phenotypic features of the original clinical cancer, offer a powerful tool for the study of tumor biology and the evaluation of anticancer drugs. Recently, we established PDTX models of colon carcinoma, and successfully evaluated a novel molecular drug [20,21]. In the present study, we evaluated the antitumor efficacy of luteolin in cMet-overexpressing PDTX models as well as in gastric cancer cell lines.

## Materials and methods

### Reagents and drugs

The antibodies against cMet, Akt and ERK, and phosphorylation-specific antibodies against phospho-Met (Y1234/1235), Akt (Ser308 and Ser473) and ERK (Thr202/Tyr204) were purchased from Cell Signaling Technology (Danvers, MA, USA). The antibodies against Her2, MMP9, Ki-67, caspase-3, cleaved caspase-3, poly(ADP-ribose) polymerase (PARP), cleaved PARP and glyceraldehyde 3-phosphate dehydrogenase (GAPDH) were obtained from Epitomics, Inc. (Burlingame, CA, USA). Horseradish peroxidase-conjugated secondary antibodies were sourced from Santa Cruz Biotechnology, Inc. (Santa Cruz, CA, USA). Luteolin was purchased from Sigma-Aldrich (St. Louis, MO, USA). LY294002 and PD98059 were obtained from Selleck Chemicals LLC (Houston, CA, USA).

### Establishment of xenografts and treatment protocol

Four-to-six-week-old female BALB/c nude mice, purchased from Shanghai Slac Laboratory Animal Corporation (Shanghai, China), were housed with regular 12-hour light/12-hour dark cycles for at least three days before use. Animal care was carried out in accordance with the Principles of Laboratory Animal Care (NIH publication #85-23, revised in 1985). All experimental protocols conducted in the present study were approved by the Institutional Animal Care and Use Committee of Zhejiang University (approval ID: SYXK[ZHE]2005-0072). Tumor specimens were obtained at initial surgery, after the patient had provided written informed consent. The patient had not received chemotherapy or radiation therapy before surgery. The tumors were diagnosed as poorly differentiated adenocarcinoma, according to WHO criteria (Additional file 1: Table S1). The PDTX xenograft models of gastric carcinoma were established as previously described [21,22]. Briefly, the tumors were implanted subcutaneously into the flanks of mice, under anesthesia with isoflurane. Growth of the xenografts was monitored at least twice-weekly by vernier caliper measurement of the length (a) and width (b) of the tumor. After reaching a volume of about 1500 mm^3^, the tumor was removed for serial transplantation.

Xenografts from the third generation (the second mouse-to-mouse passage) were used for the experiments, once the tumor volume had reached about 100 mm^3^. Mice with third generation xenografts were randomized into two groups (5–6 mice per group), to receive either luteolin (10 mg/kg) or dimethylsulfoxide (DMSO) vehicle by intraperitoneal (ip) injection daily for 1 month. Mouse weight and tumor volume were measured daily(5 mice per group). Tumor volume was calculated as (length × width^2^)/2. Relative tumor growth inhibition (TGI) was calculated using the formula: TGI = 1 - T/C, where T/C represents the relative tumor growth of luteolin-treated mice divided by the relative tumor growth of control (DMSO-treated) mice.

### Histology and immunohistochemistry

Tumor-bearing mice were anesthetized and the tumors harvested. Tumor specimens were then fixed in 4% paraformaldehyde for 12 hours and embedded in paraffin. Five-micrometer sections were cut, dewaxed, rehydrated, and stained with hematoxylin and eosin (H&E) as described previously [23]. For immunohistochemical staining, five-micrometer sections were cut, dewaxed, rehydrated, and subjected to antigen retrieval. After quenching endogenous peroxidase activity and blocking nonspecific binding sites, the sections were incubated with primary antibodies against cMet (1:100), HER2 (1:100), MMP9 (1:200) and Ki-67 (1:500) at 4°C for 12 hours. This was followed by a 30-min incubation with secondary antibody. Immunohistochemistry was performed using the streptavidin-biotin peroxidase complex method (Lab Vision, Fremont, CA, USA). The sections were observed using an optical microscope (Nikon, Tokyo Japan; 200×).

The expression of cMet was determined according to HercepTest guidelines, as follows: no membrane staining or membrane staining in <10% of tumor cells, a score of 0; faint/barely perceptible partial membrane staining in >10% of tumor cells, a score of 1+; weak-to-moderate staining of the entire membrane in >10% of tumor cells, a score of 2+; and strong staining of the entire membrane in >10% of tumor cells, a score of 3+. Scores of 0 or 1+ were considered as negative for MET overexpression, and scores of 2+ or 3+ were considered as positive [5]. For MMP9 assessment, we analyzed the staining intensity and the percentage of stained tumor cells. Staining intensity was scored as 0 (none), 1 (weak), 2 (moderate), and 3+ (strong), and the percentage was scored as 0% = 0 points, ≤25% = 1 point, 26 to 50% = 2 points, and ≥50% = 3 points. We calculated the final score by multiplying the respective scores. For Ki-67 assessment, one hundred cells were randomly selected and counted from five representative fields of each section, the percentage of stained tumor cells were calculated. All immunohistochemical slides were reviewed by two independent pathologists.

### Western blotting

Briefly, lysates for immunoblotting were prepared by adding lysis buffer (50 mM Tris–HCl [pH 7.4], 1% Nonidet P-40, 0.5% sodium deoxycholate, 150 mM NaCl, 0.02% sodium azide, and 0.1% SDS) containing protease and phosphatase inhibitors (Sigma-Aldrich, St. Louis, MO, USA) to cells or tumor tissue homogenized in liquid nitrogen. Appropriate cell and tissue protein extracts were fractionated by SDS-PAGE and electro-transferred to polyvinylidene difluoride (PVDF) membranes (Millipore, Billerica, MA, USA). After blocking for 1 h at room temperature in 5% milk in TBS-T (10 mM Tris–HCl [pH 7.5], 0.5 M NaCl, and 0.05% [w/v] Tween 20), the membranes were incubated overnight at 4°C with appropriate primary antibodies. The next day, the membranes were washed and then incubated with suitable peroxidase-conjugated secondary antibodies for 1 h at room temperature. After washing three times with TBS, the blot was soaked for 1 min in ECL™ chemiluminescent detection reagents (Millipore, Billerica, MA, USA). The membranes were then placed between two sheets of plastic wrap and exposed to film (Kodak, Rochester, NY, USA) for 30 s in a darkroom. To show equal protein loading, the blots were stripped and reprobed for peroxidase-conjugated GAPDH antibody. The experiments were repeated at least three times.

### Cell culture

MKN45, MKN28, BGC823, AGS and SGC7901 cells were obtained from the Culture Collection of the Chinese Academy of Sciences (Shanghai, China). These cell lines were passaged for fewer than 6 months after resuscitation. Cell lines were routinely cultured at 37°C in the presence of 5% CO_2_ in RPMI 1640 (Invitrogen, Carlsbad, CA, USA) supplemented with 10% FBS (Hyclone; GE Healthcare, Little Chalfont, UK).

### Cell viability assay

The effect of luteolin on cell viability was assessed using the methyl-thiazolyl-tetrazolium (MTT) assay. In brief, cells were seeded into 96-well plates at 5000 cells per well. After overnight incubation, the cells were treated with DMSO vehicle (1 μL/mL) and varying concentrations of luteolin (20, 40 and 80 μM in DMSO) for 24, 48 and 72 h. For measurement of cell growth, each well was incubated with MTT (0.5 mg/mL) for 4 h at 37°C. Afterwards, the supernatant was removed and the formazan crystals dissolved in 200 μL DMSO at room temperature. Absorbance of the solution was then measured at a 490-nm wavelength using an MRX II absorbance reader (Dynex Technologies, Chantilly, VA, USA).

### Cell apoptosis assay

The apoptosis-inducing effect of luteolin was investigated using annexin V-fluorescein isothiocyanate (FITC) and flow cytometry. Cells grown in six-well plates were treated with varying concentrations of luteolin (0–80 μM in DMSO) for 24 h. The cells were then harvested, washed twice in PBS, resuspended in binding buffer at a concentration of 1 × 10^6^ cells/mL, and mixed with 5 μL annexin V-FITC and 5 μL propidium iodide for 15 min. Stained cells were analyzed using an FC500 flow cytometer and CXP software (Beckman Coulter, Fullerton, CA, USA). The percentage of apoptotic cells was determined by the CXP software.

### In vitro migration and invasion assay

Cell migration and invasion assays were carried out as described previously [24]. For these assays, the cells were pre-starved in serum-free medium for 12 h. According to the protocol provided by the manufacturer (Millipore, Billerica, MA, USA), 900 μL of medium with 10% FBS was added into the wells of a 24-well plate, and 8-mm pore transwell inserts were plated into these wells for 1-h rehydration at 37°C . For the cell invasion assay, the upper chamber of a transwell was coated with Matrigel (BD Biosciences, San Jose, CA, USA) for 30 min at 37°C before rehydration. Starved cells were resuspended in serum-free medium at 3 × 10^5^ cells per well for the invasion assay, and 1 × 10^5^ cells per well for the migration assay, and seeded into the upper chamber. Both the upper chamber and lower chamber contained varying concentrations of luteolin (0–20 μM in DMSO). After incubation for 24 h at 37°C, cells on the upper side of the membrane were removed with cotton swabs. The cells on the lower surface of the filters were fixed with methanol and stained with 0.1% crystal violet. The numbers of migrated or invaded cells were then counted from 5 random fields under × 200 magnification.

### Statistical analysis

All values were tested for normal distribution and are expressed as mean ± SD. Differences between groups were assessed using the Student’s t-test (two-tailed). P < 0.05 was taken to indicate statistical significance. Statistical calculations were performed using SPSS 16.0 software (IBM Corporation, Armonk, NY, USA).

## Results

### Histological and molecular characterization of the PDTX models of gastric cancer

**Figure 1:**
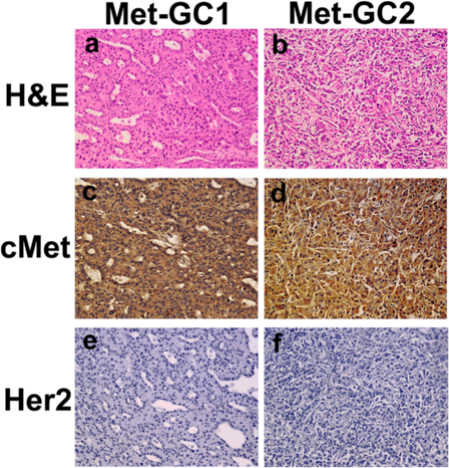
**Histological and molecular characterization of the PDTX models of gastric cancer.** a, b: H&E staining. c, d: Immunohistochemical staining for cMet. e, f: Immunohistochemical staining for Her2. Immunodetectable protein is indicated by brown staining; nuclei are counterstained blue. Original magnification, ×200.

The PDTX models of gastric cancer were successfully established, with histological examinations of H&E sections from the third generation xenografts of both Met-GC1 and Met-GC2 showing poorly differentiated adenocarcinoma consistent with the original clinical cancer (Figure 1a, b). We had previously ascertained that PDTX xenografts could retain the histopathologic and molecular characteristics of the original clinical cancer [20,21]. The expressions of cMet and HER2 were evaluated, since trastuzumab has been recommended as a treatment for patients with Her2-overexpressing gastric cancer. Immunohistochemical analysis showed that both the Met-GC1 and Met-GC2 xenograft models were positive for cMet overexpression (+++) but negative for Her2 overexpression (−) (Figure 1c–f).

### Luteolin inhibits tumor growth in PDTX models of gastric cancer

**Figure 2:**
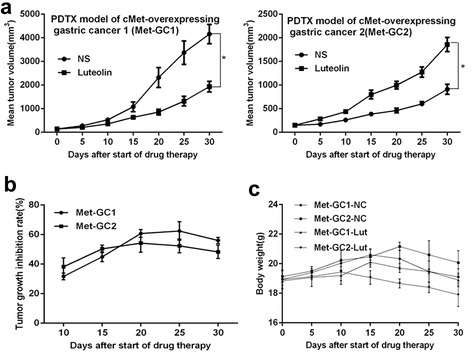
**Luteolin inhibits tumor growth in cMet-overexpressing PDTX models of gastric cancer. a**: Changes in tumor volume in Met-GC1 and Met-GC2 models of gastric cancer treated for 30 days with luteolin (10 mg/kg ip daily) or DMSO (as a vehicle control). **b**: Tumor growth inhibition rates in Met-GC1 and Met-GC2 models of gastric cancer treated with luteolin (10 mg/kg ip daily). **c**: Changes in mouse body weight in Met-GC1 and Met-GC2 models of gastric cancer treated for 30 days with luteolin (10 mg/kg ip daily) or DMSO (as a vehicle control). Data are presented as the mean ± SD. *P < 0.05 (Student’s t-test).

We next examined the effects of luteolin on growth of the PDTX models of gastric cancer. Luteolin significantly inhibited tumor growth, compared with the DMSO vehicle control, in both the cMet-GC1 and cMet-GC2 models (Figure 2a). No obvious differences were observed between the Met-GC1 and Met-GC2 models in the rate of tumor inhibition by luteolin (Figure 2b). No apparent toxicity or weight loss was observed with luteolin administration during the experimental period (Figure 2c).

**Figure 3:**
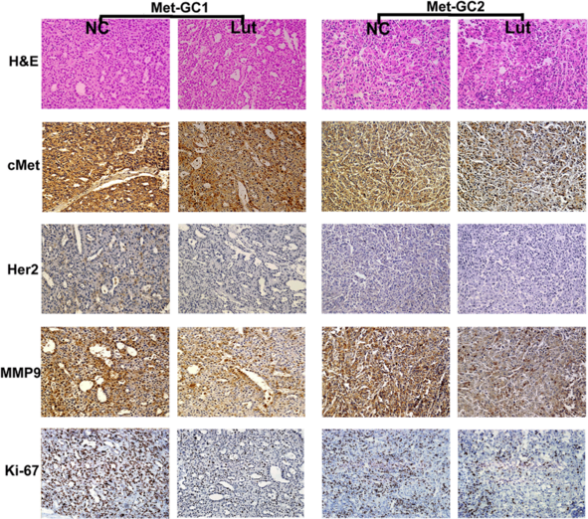
**Effects of luteolin administration on the expressions of cMet, Akt, ERK, MMP9 and Ki-67 in PDTX models of gastric cancer.** H&E staining and immunohistochemistry were undertaken after administration for 30 days of either luteolin or DMSO (as a vehicle control). Immunodetectable protein is indicated by brown staining; nuclei are counterstained blue. Original magnification, ×200.

**Figure 4:**
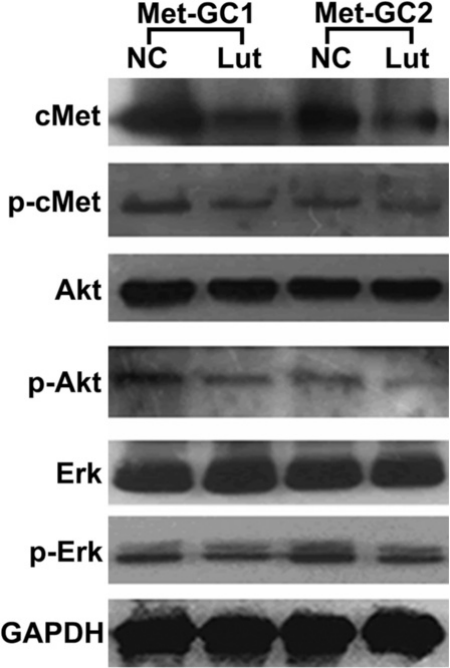
**Representative immunoblot data showing the expressions of proteins in the cMet signaling pathway.** Western blotting was employed to probe for cMet, p-cMet, Akt, p-Akt, ERK and p-ERK; GAPDH was used as a loading control.

Effects of luteolin administration on the expressions of cMet, p-cMet, Akt, p-Akt, ERK, p-ERK, MMP9 and Ki-67 in PDTX models of gastric cancer cMet-overexpression in gastric cancer is accompanied by abnormal activation of cMet signaling that contributes to tumor survival, growth and metastasis. We conducted immunohistochemical and Western blot analysis of the cMet signaling pathway. Immunohistochemistry demonstrated that the expression of cMet protein was significantly decreased in the luteolin-treated groups. The expressions of MMP9, an indicator of potential metastatic capability, and Ki-67, a proliferation maker, were also greatly decreased in the luteolin-treated groups (Figure 3, Additional file 2: Figure S1). Western blot analysis revealed down-regulated expression of cMet and phosphorylated cMet in the luteolin-treated groups. Only slight inhibition of phosphorylated ERK and Akt was observed in the luteolin-treated groups (Figure 4).

**Figure 5:**
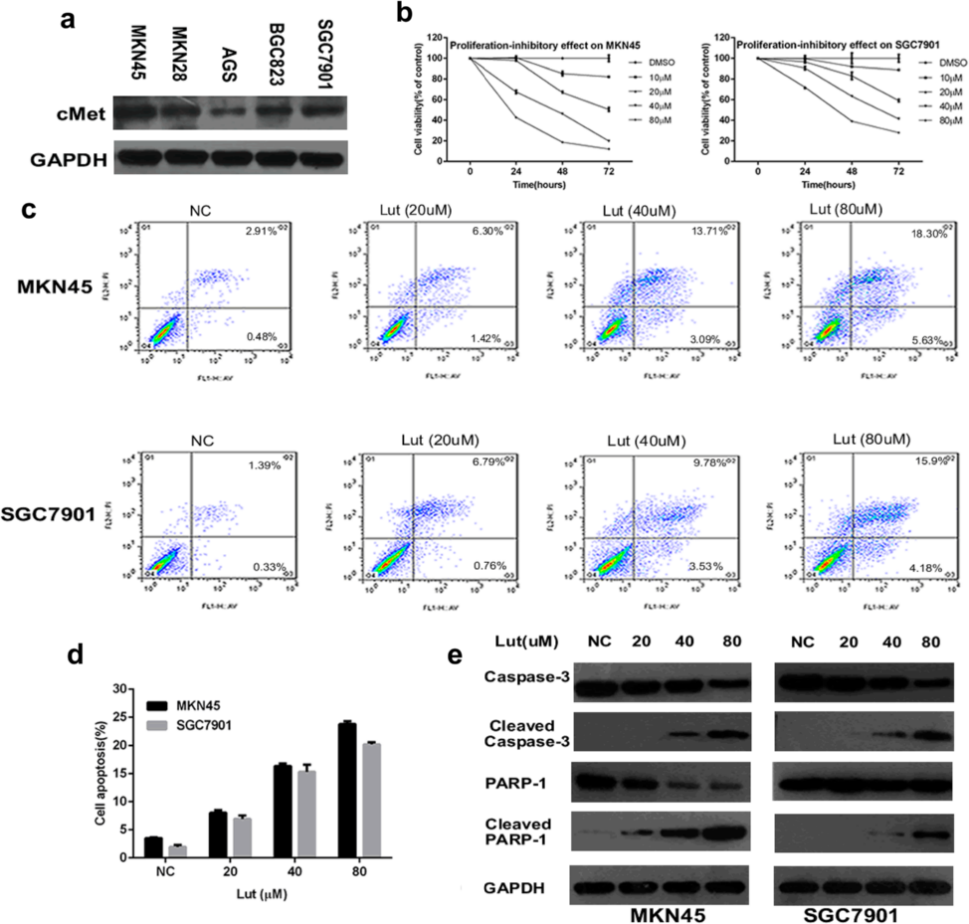
**Luteolin inhibits proliferation of MKN45 and SGC7901 gastric cancer cells. a**: Western blot analysis of cMet expression in a series of gastric cancer cell lines, including MKN45, MKN28, AGS, BGC823 and SGC7901 cells. **b**: Assessment of cell proliferation and viability, using the MTT assay, in MKN45 and SGC7901 cells treated with varying concentrations of luteolin (10–80 μM) or DMSO (1 μL/mL) for 24, 48 or 72 h. **c**: Evaluation of cell apoptosis, using flow cytometry and propidium iodide/annexin-V staining, in MKN45 and SGC7901 cells treated with varying concentrations of luteolin (10–80 μM) or DMSO (1 μL/mL) for 24 h. The gate setting distinguished between early apoptotic (bottom right), late apoptotic (top right), living (bottom left) and necrotic (top left) cells. **d**: The percentage of total apoptotic MKN45 and SGC7901 cells for varying concentrations of luteolin (20–80 μM) or DMSO (1 μL/mL), quantified from three independent experiments. **e**: Western blot analysis of cleaved caspase-3 and cleaved PARP expression after treatment with luteolin for 24 h. Data are presented as the mean ± SD. The blots shown are representative of those from at least three independent experiments.

Luteolin inhibits proliferation and induces apoptosis of MKN45 and SGC7901 cells.To further investigate the possible mechanisms involved in the anti-tumor effects of luteolin in cMet-overexpressing gastric cancer, we first examined cMet expression in a series of gastric cancer cells, including MKN45, MKN28, AGS, BGC823 and SGC7901 cells. On the basis of these data, MKN45 and SGC7901 cells, which showed high expression of cMet, were selected for further study (Figure 5a). The MTT assay demonstrated that luteolin markedly inhibited the growth of MKN45 and SGC7901 gastric cancer cells in a concentration- and time-dependent manner (Figure 5b). To determine whether luteolin-induced cell death in MKN45 and SGC7901 cells was via an induction of apoptosis, we evaluated cell apoptosis using flow cytometry analysis, with propidium iodide and annexin-V staining. The data indicated that luteolin administration caused apoptosis in a concentration-dependent manner (Figure 5c). The percentage of apoptotic MKN45 and SGC7901 cells increased from 8.0% and 6.9%, respectively, at 20 μM luteolin to 23.7% and 20.2%, respectively, at 80 μM luteolin (Figure 5d). Western blot analysis revealed increased expression of cleaved caspase-3 and cleaved PARP (indicators of apoptosis) (Figure 5e).

**Figure 6:**
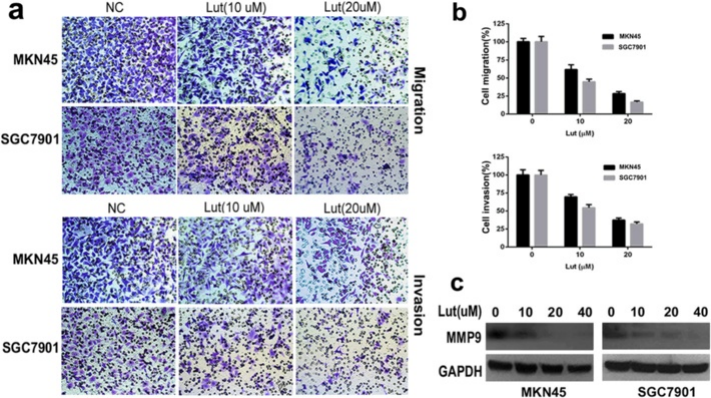
**Inhibitory effects of luteolin on MKN45 and SGC7901 cell migration and invasion. (a)** Representative images showing MKN45 and SGC7901 cell migration and invasion. Cells were seeded in transwell plates, incubated with 0–20 μM luteolin for 24 h, and then fixed and stained with 0.1% crystal violet. **(b)** Cells (blue in color) on the lower surface were counted in triplicate, in three independent experiments, using a light microscope. **(c)** MMP9 expression evaluated by Western blotting. Data are presented as the mean ± SD. Original magnification, ×200.

Luteolin inhibits the migration and invasion of MKN45 and SGC7901 cells.Low concentrations of luteolin (0–20 μM) administered for 24 h did not affect the viability of MKN45 and SGC7901 cells. However, as shown in Figure 6b, administration of low concentrations of luteolin resulted in a concentration-dependent inhibition of migration and invasion of both MKN45 and SCG7901 cells. Since MMP9 expression was found to be decreased in Met-GC1 and Met-GC2 models treated with luteolin (see above), the expression of MMP9 was also evaluated in MKN45 and SGC7901 cells treated with luteolin for 24 h. As shown in Figure 6c, luteolin also down-regulated MMP9 expression in both these gastric cancer cell lines. As MMP9 plays an important role in tumor invasion, the down-regulation of MMP9 may be involved in the inhibition of invasiveness by luteolin.

### Luteolin down-regulates cMet/Akt/ERK signaling in MKN45 and SGC7901 cells

**Figure 7:**
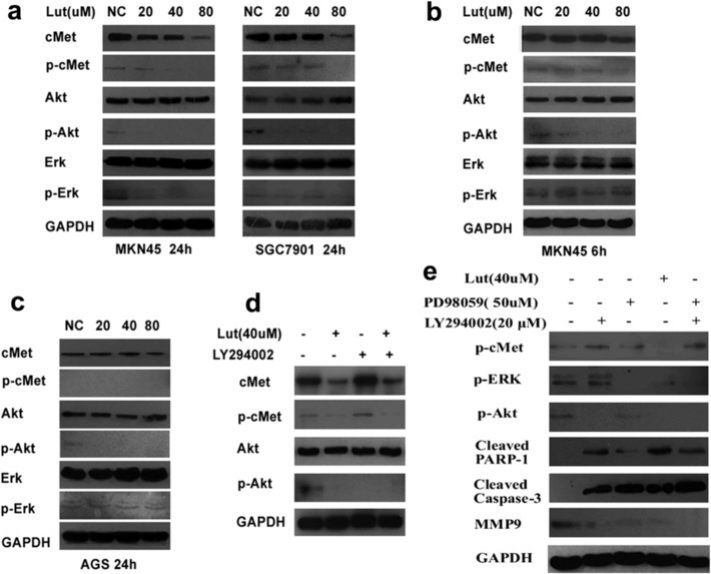
**Effects of luteolin on cMet/Akt/ERK signaling pathways (a)** **Western blot analysis of MKN45 and SGC7901 cells treated with varying concentrations of luteolin (0–80 μM) for 24 h.** **(b)** Western blot analysis of MKN45 cells treated with varying concentrations of luteolin (0–80 μM) for 6 h. **(c)** Western blot analysis of AGS cells treated with varying concentrations of luteolin (0–80 μM) for 24 h. **(d)** cMet/Akt/ERK status in MKN45 cells incubated for 24 h with LY294002 (a PI3K inhibitor; 20 μM), luteolin (40 μM) or both. **(e)** Western blot analysis of the possible involvement of Akt/ERK in cell apoptosis and invasiveness. MKN45 cells were incubated for 24 h with either LY294002 (20 μM), PD98059 (50 μM), luteolin (40 μM), or a combination of LY294002 and PD98059. GAPDH was used as a loading control. The blots shown are representative of those from at least three independent experiments.

To further examine the involvement of the cMet signaling pathway in the anti-proliferative and anti-invasive actions of luteolin, we treated cells with various concentrations of luteolin (0–80 μM) for 24 h, and performed immunoblotting assays. As shown in Figure 7a, luteolin caused concentration-dependent reductions in total cMet protein and phosphorylated cMet levels in MKN45 and SGC7901 cells. Phosphorylated Akt and ERK, downstream signaling molecules of cMet, were also down-regulated. At a luteolin concentration of 20 μM, phosphorylated Akt was significantly down-regulated despite no obvious change in the phosphorylation of cMet (Figure 7a), indicting that Akt down-regulation preceded cMet down-regulation. To examine this hypothesis, Western blot analysis was carried out in MKN45 cells treated with luteolin for 6 h, with reference to a previous study [19]. As shown in Figure 7b, at a concentration of 40 μM, luteolin significantly suppressed phosphorylated Akt but did not affect cMet phosphorylation. Furthermore, when AGS cells, which show low cMet expression, were treated with various concentrations of luteolin (0–80 μM) for 24 h, Western blot assays showed that luteolin significantly suppressed phosphorylated Akt with no obvious change in the phosphorylation of cMet and ERK (Figure 7c). Hence, the down-regulation of phosphorylated Akt appeared to be independent of cMet status, even occurring ahead of the change in cMet. Therefore, we further investigated whether the decrease in phosphorylated Akt led to the down-regulation of phosphorylated cMet. To do this, we used LY294002, a PI3K inhibitor, to determine whether prolonged inhibition of Akt phosphorylation resulted in a reduction in cMet phosphorylation. The data revealed that the administration of LY294002 to MKN45 cells for 24 h was without effect on cMet phosphorylation, despite an inhibition of phosphorylated Akt (Figure 7d). Further consideration of the data presented in Figure 7a, b, c suggested that the decrease in cMet expression mirrored the reduction in cMet phosphorylation. Thus, it was likely that the reduction in phosphorylated cMet was the result of a down-regulation in cMet expression. Together, the data indicate that luteolin could down-regulate total cMet and phosphorylated cMet, and inhibit the activity of downstream Akt and ERK signaling.

### Involvement of Akt and ERK in the regulation of caspase-3, PARP-1 and MMP9

We next assessed the contributions of Akt and ERK in the effects of luteolin to induce apoptosis and inhibit invasiveness. MKN45 cells were harvested for Western blot analysis after exposure for 24 h to either the PI3K inhibitor, LY294002 (20 μM), the ERK inhibitor, PD98059 (50 μM), luteolin (40 μM), or the combination of LY294002 and PD98059. As shown in Figure 7e, the administration of LY294002 or PD98059 inhibited p-Akt or p-ERK, respectively. Combination treatment with LY294002 and PD98059 suppressed p-Akt and p-ERK simultaneously, similar to the effect of luteolin. Furthermore, the apoptosis-related proteins, cleaved caspase-3 and cleaved PARP-1, were both up-regulated in all four treatment groups (LY294002, PD98059, luteolin, or LY294002 plus PD98059), while MMP9 was down-regulated in all four treatment groups, and particularly in the LY294002 plus PD98059 group. Thus, both PD98059 and LY294002 were able to mimic the effects of luteolin on the regulation of caspase-3, PARP-1 and MMP9. Since caspase-3, PARP-1 and MMP9 are involved in cell apoptosis and invasion, we suggest that the down-regulation of cMet/Akt/ERK is, at least in part, involved in the effects of luteolin to promote apoptosis and inhibit invasiveness.

## Discussion

cMet-overexpressing gastric cancer is associated with advanced disease stage and poor prognosis [6,7]. The aggressive nature of cMet-overexpressing gastric cancer and the lack of effective therapeutic options make this cancer particularly clinically challenging, so it is crucial that new therapies are identified. However, research in this area has been hampered by a lack of clinically relevant experimental models. It is well established that PDTX models better retain the histopathologic, genetic and phenotypic features of the original tumor than conventional cell-implanted xenografts [22]. Hence, PDTX models have been increasingly used as a tool for the preclinical assessment of anticancer drugs. Our group has previously established PDTX models of colon carcinoma, and successfully evaluated a novel VEGF-targeted agent [20,21].

In this study, we evaluated the antitumor effects of luteolin in cMet-overexpressing PDTX models of gastric cancer. To our knowledge, this is the first study of luteolin in PDTX models. Our results showed that luteolin exerted a significant antitumor effect in these models of gastric cancer. An in vitro study further revealed that luteolin greatly inhibited the proliferative and invasive activity of MKN45 and SGC7901 gastric cancer cells, which highly expressed cMet. Immunohistochemical data demonstrated that luteolin greatly reduced the expressions of cMet, MMP9 and ki-67 (Figure 3); consistent with this, the in vitro study indicated that luteolin significantly down-regulated cMet signaling and MMP9 (Figures 6, 7). Together, these data suggest that luteolin may be a candidate therapeutic option for cMet-overexpressing gastric cancer.

Hyperactivation of the cMet signaling pathway has been frequently observed in cMet-overexpressing cancer, and reported to be associated with tumor survival, growth, angiogenesis and metastasis [3,4,7]. Previous studies have indicated that luteolin exerts its anti-tumor activity by affecting numerous biochemical pathways critical for the regulation of cell survival, apoptosis, angiogenesis and metastasis, including PI3K/Akt, NF-κB, MAPKs, MMPs and E-cadherin [14-18]. Lee et al. demonstrated that luteolin suppressed HGF-induced phosphorylation of c-Met in human hepatoma HepG2 cells. Coleman et al. further revealed that luteolin post-transcriptionally down-regulated c-Met expression independently of proteosomal/lysosomal degradation in DU145 prostate cancer cells. Recently, Wu et al. reported that luteolin can induce apoptosis by up-regulating miR-34a in gastric cancer cells [25]. Anyway, further investigations were still needed to elucidate the possible mechanisms of antitumor effects of luteolin in gastric cancer. In the present study, we observed that luteolin decreased the expression and phosphorylation of cMet in both cMet-overexpressing gastric tumor tissue and gastric cancer cells with high cMet expression (Figures 3, 4, 7a). We further revealed that downstream Akt and ERK signaling was also down-regulated in MKN45 and SGC7901 cells. Interestingly, luteolin also caused a down-regulation of Akt without affecting the activity of cMet and ERK (Figure 7b, c), suggesting that the inhibition of Akt by luteolin could be independent of cMet. We also showed that luteolin-induced down-regulation of phosphorylated Akt occurred ahead of the effects on cMet. We then confirmed that prolonged inhibition of phosphorylated Akt had no influence on phosphorylated cMet (Figure 7c). Further analysis of the results in Figure 7a, b, c suggested that the decrease in cMet expression mirrored the reduction in cMet phosphorylation. This finding implies that luteolin can down-regulate total cMet and phosphorylated cMet, and inhibit downstream Akt and ERK signaling, while also inhibiting Akt activity independently of cMet.

Previous research in different cell types has demonstrated that Akt and ERK signaling play a central role in the regulation of cell survival, proliferation and metastasis [26-28]. Activation of the Akt and ERK pathway is common in cMet-overexpressing cancer [4], and this activity can lead to a prevention of apoptosis [29,30]. In this study, we found that luteolin promoted the apoptosis of MKN45 and SGC7901 cells in a concentration-dependent manner, with activation of caspase-3 and PARP (Figure 5). As mentioned above, luteolin was able to down-regulate phosphorylated Akt and ERK. We further observed that both PD98059 (an ERK inhibitor) and LY294002 (an Akt inhibitor) could mimic the effects of luteolin on the activation of caspase-3 and PARP-1. Previous investigations have also shown that LY294002 or PD98059 could inhibit the growth of gastric cancer cells and induce apoptosis [31-33]. Based on these findings, we suggest that luteolin may promote apoptosis partly via the down-regulation of phosphorylated Akt and ERK.

MMP9, which can degrade collagen IV, plays an important role in cancer metastasis [34]. It has been reported that MMP9 correlates with the invasion, metastasis and angiogenesis of gastric cancer [33,35]. Several studies have indicated that Akt and ERK regulate the expression of MMPs [35,36]. Previous research has demonstrated that the inhibition of Akt by LY294002 inhibited cancer cell invasion and down-regulated MMP9 expression [37,38]. In addition, blocking the ERK1,2 pathway with a selective chemical inhibitor, PD98059, could also down-regulate MMP9 [39]. In the present study, we found that MMP9 was down-regulated in luteolin-treated tumor tissue and cancer cells (Figures 3, 6c). Significant inhibitions of migration and invasion were also observed in the in vitro study (Figure 6). To determine whether down-regulation of phosphorylated Akt and ERK affected the expression of MMP9, we treated MKN45 and AGS cells with LY294002 and/or PD98059: we observed that LY294002 or PD98059 could down-regulate MMP9, an effect similar to that of luteolin (Figure 7d). Therefore, down-regulation of MMP9 may be involved in the inhibition of invasiveness by luteolin.

Previous investigations have also reported that luteolin can affect various receptors, such as EGFR, Her2 and the androgen receptor [11,12,40]. In the present study, we examined the cMet and HER2 status of the PDTX models. The expression of EGFR was also examined, and found to be Met-GC1 (−) and Met-GC2 (+) (immunohistochemistry; data not shown). However, the in vitro study only examined the cMet status of MKN45 and SGC7901 cells. In effect, there is a considerable amount of HER2 and EGFR expression in MKN45 and SGC7901 cells, so we cannot exclude crosstalk effects from other pathways. Moreover, we observed a decrease in phosphorylated Akt in luteolin-treated MKN45, SGC7901 and AGS cells. Although the present study has not definitively established the underlying mechanisms, a possible explanation is that luteolin can target Akt either directly or through other pathways. Further studies are merited to explore these possibilities. In addition, although we successfully established two cMet-overexpressing gastric cancer models in this study, we are now establishing more PDTX models, since it is essential that clinically reliable experimental systems are developed to facilitate the discovery of novel therapeutic options for cMet-overexpressing gastric cancer.

## Conclusions

In summary, our findings strongly complement the current knowledge concerning the treatment of cMet-overexpressing gastric cancer. Our data demonstrate, for the first time, that luteolin exerts marked antitumor effects in cMet-overexpressing PDTX models of gastric cancer, at least in part via down-regulation of cMet/Akt/ERK signaling. These findings indicate that luteolin may act as a potential therapeutic option for cMet-overexpressing gastric cancer. Further study focusing on the role of luteolin in cMet-overexpressing gastric cancer will provide more detailed insights for the antitumor effects of luteolin in the future.

## Additional files

Additional file 1: Table S1. Clinical characteristics of the patients used for developing PDTX models. Additional file 2: Figure S1. Quantitative analysis of Ki-67 expressions after the administration of luteolin. Quantitative analysis of Ki-67 expression was determined as described in Materials and Methods. Data are presented as the means ± SD. *P < 0.05 (Student’s t-test).
