# Supplementary material for: Luteolin exerts a marked antitumor effect in cMet-overexpressing patient-derived tumor xenograft models of gastric cancer
Source: J Transl Med. 2015 Feb 1;13:42. doi: 10.1186/s12967-015-0398-z (PMC4320638; doi:10.1186/s12967-015-0398-z)
Supplement: Additional file 1: Table S1. — Clinical characteristics of the patients used for developing PDTX models. [file 12967_2015_398_MOESM1_ESM.docx]

|  |  |  |  |  |  |  |  |  |  |
| --- | --- | --- | --- | --- | --- | --- | --- | --- | --- |
| **Supplement table1. Clinical characteristics of the patients used for developing PDTX models** | | | | | | | | | |
| Models | Sex | Age(years) | Serum CEA | Surgery | Locations | Histological type | TNM Stage | Patient status | Survival time (months) |
| Met-GC1 | Male | 56 | Normal | Subtotal gastrectomy | Body | Poorly differentiated | T3N2M0(IIIA) | Death | 24 |
| Met-GC2 | Female | 60 | Normal | Subtotal gastrectomy | Antrum | Poorly differentiated | T3N3M0(IIIB) | Death | 17 |
|  |  |  |  |  |  |  |  |  |  |
